# Fraudulent Uses of Sulphuric Acid

**Published:** 1850-01

**Authors:** H. Letheby

**Affiliations:** London, Lecturer on Chemistry at the Medical College of the London Hospital


### CHEMISTRY.

*Fraudulent uses of Sulphuric Acid.*—By H. Letheby, M.B., London, Lecturer on Chemistry at the Medical College of the London Hospital. —Sulphuric acid and the sulphates are not uncommonly employed as sophisticating agents; for example, alum and sulphuric acid are used for the purpose of increasing the acidity of wines and vinegar; sulphuric acid is employed in the manufacture of a saccharine compound, which is used in the adulteration of cane sugar; and alum, plaster of Paris, and sulphate of copper are frequently resorted to for the purpose of whitening and increasing the sponginess of bad bread. All these adulterations are matters of very serious moment both to you and the public, and the discussion of them is, in my opinion, very properly suited to this course of lectures. I shall, therefore, direct your attention to them in a very pointed manner.

*All the Vinegars* of English commerce contain free sulphuric acid, inasmuch as the vinegar manufacturers are permitted by law to employ a certain portion, viz: the one-thousandth part by weight of oil of vitriol, for the purpose of checking the putrefaction to which the gluten of the liquid is subject: but, as Dr. Ure very properly remarks, "this is a miserable shift, or pretended necessity, in the present advanced state of organic chemistry. It offers, besides, an easy source of fraud, since neither the retailers nor consumers of the article are competent to distinguish how much of the sourness is derived from the mild fermented acid, and how much from the corrosive mineral. All the pickles in which our *bourgeoisie* so much delight are polluted by the same sophistication. Not long ago," continues Dr. Ure, "a sample of vinegar was submitted to me for examination, said to be that supplied by contract to the British Navy. I found it to contain little more than half the fair amount of *proof* vinegar, with much gluten, and a copious supplement of oil of vitriol." Here, also, is a sample of cheap vinegar, which I have just obtained from one of the retailers in this neighborhood, and you will notice that it yields a copious precipitate with a soluble salt of baryta; it contains, in fact, as much as 0·65 per cent. of sulphuric acid, and a fluid ounce of it yields very nearly nine grains and a half of sulphate of baryta, in place of one grain and fourteen hundredths which are allowed by law.

On the Continent, also, manufacturers are accustomed to adulterate vinegar, and many varieties of low red wines, with alum or sulphuric acid; and the fraud has become so serious, that chemists have thought it necessary to inquire into the matter, in the hope of being able to point out a ready mode of detecting the imposition. To this end Orfila directs that the suspected liquid should be agitated with ether; and, after standing for a short time, the etherial solution is to be decanted and tested for free sulphuric acid. It does not appear, however, that this mode of proceeding is a very satisfactory one; for, as I have already informed you, upon the authority of Dr. Christison, ether will not remove sulphuric acid from an aqueous solution. Guibourt, in fact, has put this to the test of actual experiment in the case of one of the liquids in question, and he has drawn from Orfila an admission that ether will only take up a trace of the poison. Lassaigne, also, states that, in an examination conducted by MM. Ossian Henri, Bayard, and himself it was not possible to separate, by the action of sulphuric ether, four or five-thousandths of sulphuric acid added to red wine, and, consequently, that this method of operating would not answer for the detection of the impurity.

In the London Pharmacopœia, it is directed that the quantity of sulphate of baryta precipitated from a fluid ounce of vinegar by the addition of a soluble salt of baryta should not exceed 1.14 grains. But this rule, however applicable it may be to the composition of malt vinegar, cannot be applied either to wines, or the vinegars obtained therefrom; for grape juice contains very variable proportions of alkaline and earthy sulphates,—salts which will be sure to vitiate the results of the inquiry. It is necessary, therefore, under these circumstances, to proceed in a different manner.

MM. Lassaigne, Ossian Henri, and Bayard, inform us that they have, after many failures, been enabled to decide on a re-action, which is so delicate, that it will discover the presence of a one-thousandth and a half of free sulphuric acid in red wine. They say that when a piece of paper which has been touched with pure wine is dried at a gentle heat, the spotted portion is unaltered; whereas, paper which has been moistened with wine, to which a very small quantity of sulphuric acid has been added, reddens, and becomes brittle and friable between the fingers, when slightly rubbed, before the white paper becomes at all coloured.

Pure wine, to which nothing has been added, leaves by spontaneous evaporation a violet blue spot; whereas, wine to which a very small quantity of sulphuric acid has been added, (two to three thousandths) gives by drying a rose-colored spot.

On examining into the sensibility of this simple process, the authors found that they were enabled to detect, by its means, one thousandth of sulphuric red wine.

The paper most proper for the experiment is common glazed paper, containing starch or fecula. This kind of paper is well known in commerce, and it is easy to discover it by the blue color which it assumes when moistened with an aqueous solution of iodine.

Now, although the re-action is a very delicate one, yet I prefer to operate in the following manner. Take a given portion of the suspected fluid (wine or vinegar,) evaporate it in a water-bath until it acquires a syrupy consistency, then treat it with strong spirit of wine, filter it; and precipitate the sulphuric acid contained therein by means of chloride of barium and a little nitric acid. In the course of this operation the alcohol will take up the free sulphuric acid, and will leave the neutral sulphates untouched.

Another, but not equally successful mode of operating is the following. Take two equal portions of the suspected liquid. Precipitate the sulphuric acid contained in one of them, and weigh the product. Evaporate the other portion to dryness, ignite it, re-dissolve the residue in water, filter it, and precipitate as before. The weight of this product indicates the amount of combined sulphuric acid present, while the weight of the former represents the entire quantity of acid (free and combined) which existed in the liquid. Upon subtracting, therefore, the weight of the one from that of the other, we can easily determine how much sulphuric acid had been fraudulently added to the liquid.

Again : *Bread* is often adulterated with metallic and earthy sulphates for the purpose of making it look white and spongy. Upon the Continent, especially in the north of France and in Belgium, public attention has been long called to the fact, that bakers are accustomed to employ a solution of blue vitriol (sulphate of copper) for this purpose ; and M. Kuhlman, formerly Professor of Chemistry, at Lille, has collected a great number of facts which bear upon this subject. They were published under the direction of the Central Council of Salubrity of the Department du Nord; and although I think it right to allude to them here, yet I shall postpone their consideration until we come to treat of the metal which constitutes the chief ingredient of the poison.

From Kuhlman's researches it appears, that sulphate of copper exerts a very energetic and peculiar action on the fermentation of bread. He states that the addition of one grain of this salt to three pounds of dough will cause it to rise to a much greater extent than it would otherwise do, and thereby make the bread look white and more vesicular.

Alum is known to operate upon dough in a similar manner ; for "it keeps water and rises well," as bakers express it, and so enables them to set off a very inferior and otherwise unsaleable flour. In the "Encyclopædia Britannica," and in the Dictionaries published by Dr. Ure, I find the following statement :

" In London, where the goodness of bread is estimated entirely by its whiteness, it is usual with those bakers who employ flour of an inferior quality, to add as much alum as common salt to the dough ; or, in other words, the quantity of salt added is diminished one half, and the deficiency supplied by an equal weight of alum. This improves the look of the bread very much, rendering it whiter and firmer." "Now," says Dr. Urie, " we may hence infer, that the full allowance here assigned of two and a quarter pounds of alum for every two pounds and a quarter of salt, will be adopted in converting a sack of flour into loaves. But as a sack of flour weighs 280 pounds, and furnishes, on an average, 80 quartern loaves, we have 2¼ pounds divided by 80,—that is, 197 grains as the quantity of alum present in a quartern loaf. And as I need hardly tell you, it is a very serious thing for a gentleman or lady of sedentary habits, or infirm constitution, to have the digestive process daily vitiated by damaged flour, whitened with 197 grains of alum per quartern loaf. Acidity of stomach, indigestion, flatulence, headaches, palpitation, costiveness, and urinary calculus, may be the probable consequences of the habitual introduction of so much acidulous and acescent matter."

My own experience is quite confirmatory of another observation made by Dr. Ure, who says, "I have made many experiments on bread, and have found the proportion of alum very variable. Its quantity seems to be proportional to the badness of the flour; and hence, when the best flour is used, no alum need be introduced.

Plaster of Paris is another agent which is sometimes used for the sophistication of bad flour. I here show you a specimen of ship-biscuit, furnished by a cheap baker to a trading captain. It contains 5·5 per cent. of sulphate of lime, which has, doubtless, been introduced far the purpose of increasing the weight and improving the appearance of a bad article.

All these adulterations may be recognized by digesting the bread after it has become very stale, in distilled water, and testing the solution with nitric acid and baryta for sulphuric acid. Good bread scarcely affords a cloudiness with this re-agent, and the precipitate so obtained is too small to be weighed. Again, the last-named adulteration may be discovered by igniting the bread and weighing the residue. Good flour or bread will not yield more than two per cent. of ash, the greater portion of which is soluble in about ten drops of water.

The last point to which I shall direct your attention before I leave this subject, is the fact, that cane sugars are frequently adulterated with a low kind of saccharine product, obtained by the action of dilute sulphuric acid on starch. This sugar, which is identical in composition with grape sugar, is recognized by its granular or powdery appearance. In fact, it does not possess a crystalline structure; and hence it is devoid of that sparkling character which is possessed by cane, maple, and beet root sugars. I am not prepared to tell you that the substance in question is positively hurtful to the human body, but I am in a condition to say, that the purpose to which it is commonly applied is injurious to the fair dealer, and is a gross fraud upon the public. Several chemical reactions have, from time to time, been referred to as a means of distinguishing the presence of glucose in the sugars of commerce, but in my opinion there are but two of them which possess any practical value:—1st. It was noticed by Mr. Moore, and his observation has been verified by Chevalier and others, that starch or granular sugar acquires a deep brown color when it is boiled with liquor potassæ, a reaction which is not manifested by any other species of saccharine matter. To employ this test, therefore, you take about ten grains of the suspected sugar, and heat it in a drachm of liquor potassæ; if the liquor acquire a deep coffee brown color, it indicates the presence of this adulterating agent.

The second reaction was first noticed by M. Trommer. It is dependent on the property which glucose or starch sugar has of reducing the hydrated oxide of copper; and, to make this reaction manifest, you proceed as follows:—Take about ten grains of the suspected sugar, and pour upon it a liquid consisting of six drops of a saturated solution of sulphate of copper, and ʒij. of liquor potassæ. Heat this mixture in a test tube; and, if granular or starch sugar be present, the solution will instantly acquire a bright orange color at the point where the flame touches the tube; and gradually, as the heat is propagated through the liquor, the whole contents of the tube will change color, and ultimately become of a deep brown tint.

Starch sugar, also, frequently contains a considerable portion of plaster of Paris, the residue of the materials employed in its manufacture; hence, if you digest such a sugar in strong alcohol, the saccharine matter will be taken up, and the sulphate of lime left behind in the form of a white powder.

By adopting one or other of these means you will have no difficulty in discovering the fraud in question.—*Med. Times.*

